# Supplementary material for: Pyramiding QTLs controlling tolerance against drought, salinity, and submergence in rice through marker assisted breeding
Source: PLoS One. 2020 Jan 7;15(1):e0227421. doi: 10.1371/journal.pone.0227421 (PMC6946594; doi:10.1371/journal.pone.0227421)
Supplement: S1 File — (PDF) [file pone.0227421.s001.pdf]

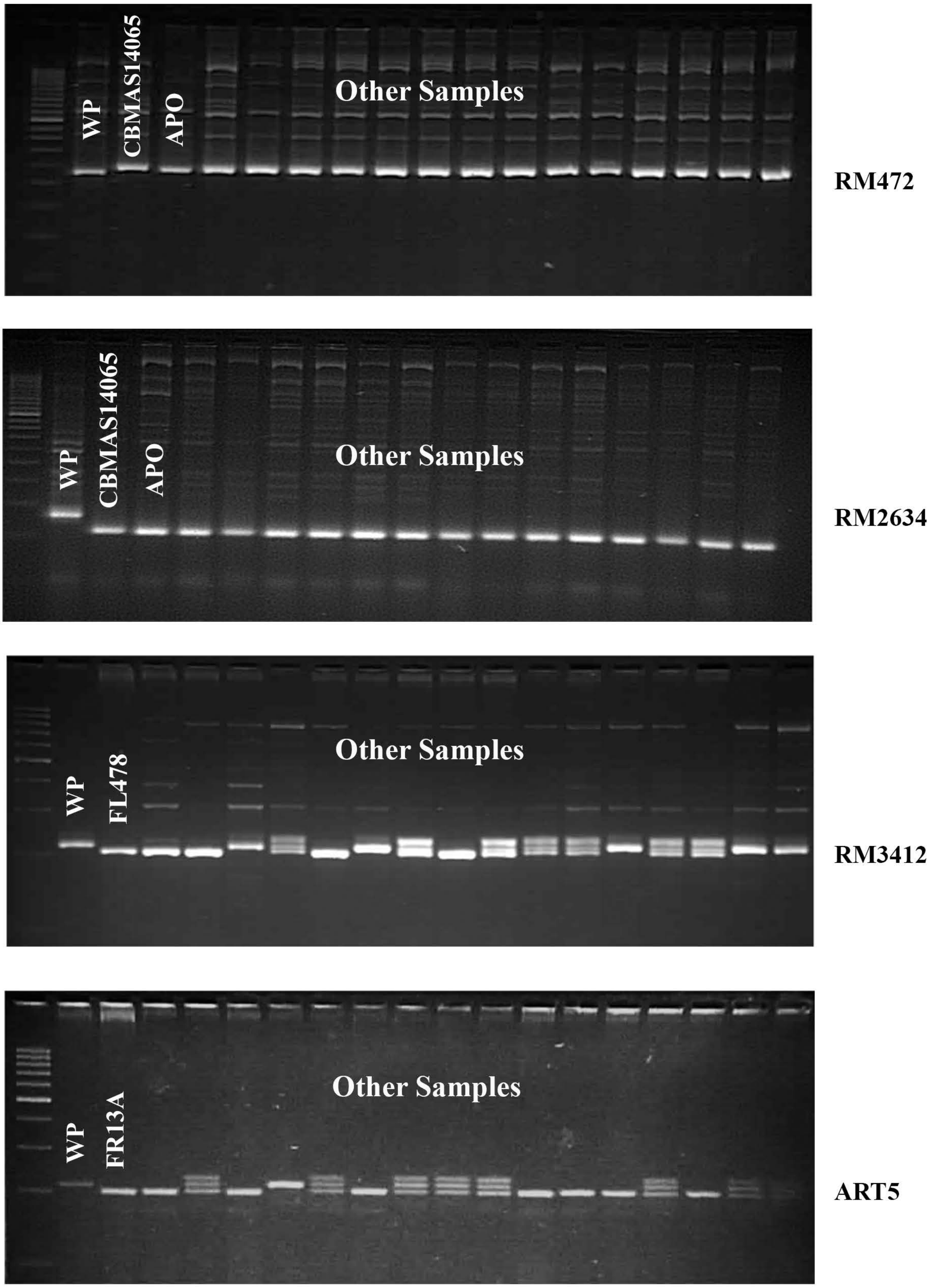

Fig. 2. Parental polymorphism for the markers linked to the target

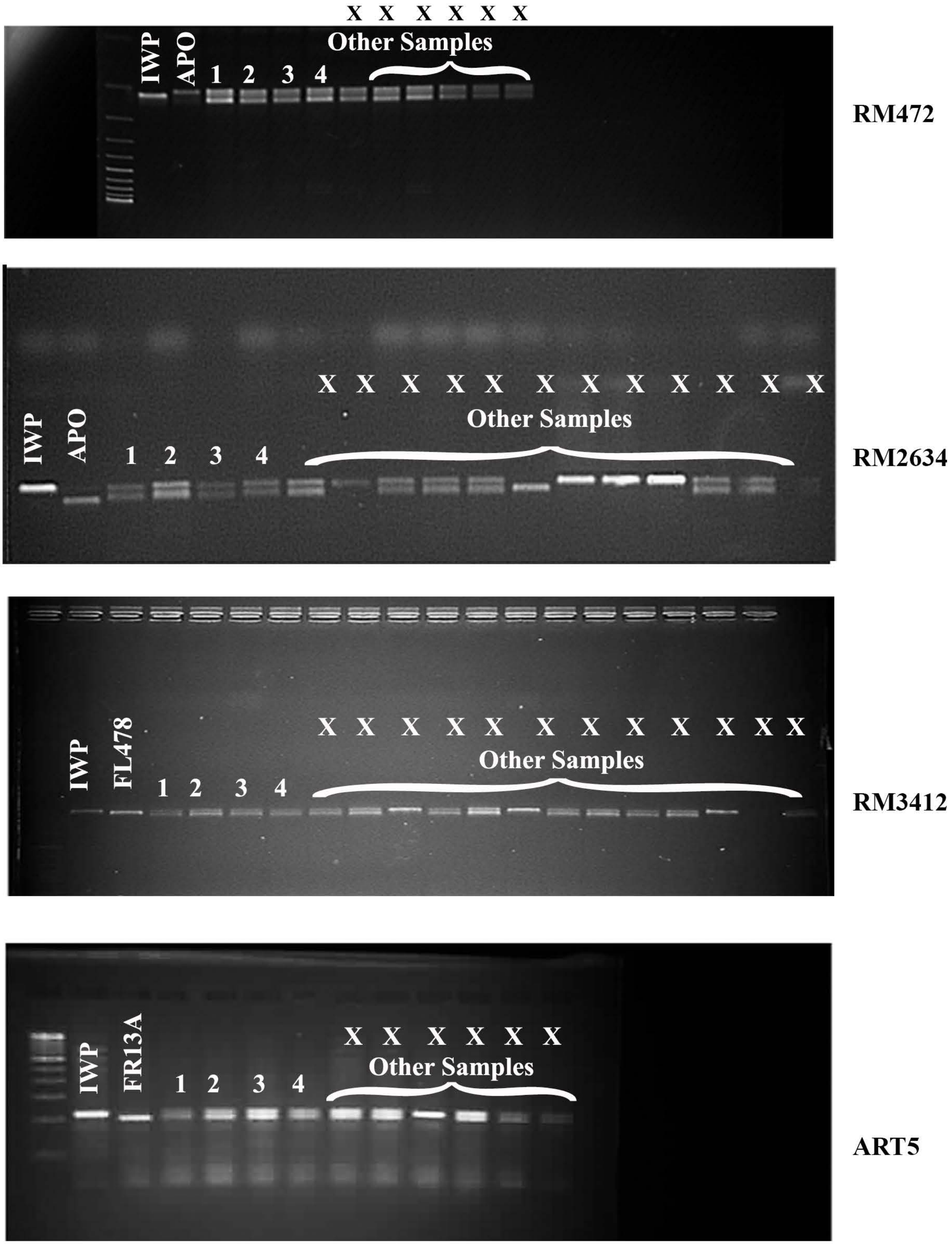

Fig 3. Foreground selection of BC1F1 progenies using markers linked to target traits
