# Supplementary material for: Pyramiding QTLs controlling tolerance against drought, salinity, and submergence in rice through marker assisted breeding
Source: PLoS One. 2020 Jan 7;15(1):e0227421. doi: 10.1371/journal.pone.0227421 (PMC6946594; doi:10.1371/journal.pone.0227421)
Supplement: Attachment — Submitted filename: Response to Reviewers Comment.docx [file pone.0227421.s002.docx]

**Author Responses to Questions**

| S.No | Reviewer’s comments | Responses to Reviewer’s Comments |
| --- | --- | --- |
| 1 | Authors have not made all data underlying the findings in their manuscript fully available | Thanks. Now, we have submitted the required raw data files as “Supporting Information” to the journal. |
| 2 | The manuscript is not written in standard English? | I wish to inform that the manuscript is edited by a Professional language Editing firm (M/s. Editage) as suggested by the Editorial Board. |
| 3 | The authors have employed a set of microsats. These are effective in many breeding applications because they are cheap and easy to score. It would however be nice to have whole genome re-sequencing of the BCI lines to understand the scale of introgression from the resistance donor parents, to understand their footprint across the rice genome. This is not so much a criticism of this paper, but a suggestion for future work. | Thanks for your comments. For now, we have used sufficient number of SSR markers for assessing the recovery of recurrent parent. Definitely we will consider the reviewer’s comments in future when we analyze the NILs. |
| 4 | The authors have also not gone to great lengths to characterize the agronomic characteristics of the BCI lines. Although we are told they have the same cooking and quality characteristics as the elite parent, no data to this effect is shown. | We respect the suggestions made by the reviewer. We have carried out extensive cooking quality tests in the parents and back cross progenies. Results are included in the manuscript. Back cross progenies were found to possess on par cooking quality traits as that of their recurrent parent |
| 5 | The manuscript is largely clearly written. I would encourage the authors to do another round of editing as a matter of good habits in the major revision phase. But I did not notice any clear typos, or issues with English grammatical usage such as tenses matching or proper article choice. | Thanks to the reviewer. The manuscript was subjected to thorough language editing by a professional firm ‘Editage” (suggested by the editorial board) in the first round itself. |
